# Impaired and facilitated functional networks in temporal lobe epilepsy^☆^

**DOI:** 10.1016/j.nicl.2013.06.011

**Published:** 2013-06-25

**Authors:** Luigi Maccotta, Biyu J. He, Abraham Z. Snyder, Lawrence N. Eisenman, Tammie L. Benzinger, Beau M. Ances, Maurizio Corbetta, R. Edward Hogan

**Affiliations:** a Department of Neurology, Washington University, St. Louis, MO, USA; b National Institute of Neurological Disorders and Stroke, National Institutes of Health, Bethesda, MD, USA; c Department of Radiology, Washington University, St. Louis, MO, USA; d Department of Neurological Surgery, Washington University, St. Louis, MO, USA; e Department of Anatomy and Neurobiology, Washington University, St. Louis, MO, USA

**Keywords:** Epilepsy, Temporal lobe, Hippocampus, Insula, fMRI, Functional connectivity

## Abstract

How epilepsy affects brain functional networks remains poorly understood. Here we investigated resting state functional connectivity of the temporal region in temporal lobe epilepsy. Thirty-two patients with unilateral temporal lobe epilepsy underwent resting state blood-oxygenation level dependent functional magnetic resonance imaging. We defined regions of interest *a priori* focusing on structures involved, either structurally or metabolically, in temporal lobe epilepsy. These structures were identified in each patient based on their individual anatomy. Our principal findings are decreased local and inter-hemispheric functional connectivity and increased intra-hemispheric functional connectivity ipsilateral to the seizure focus compared to normal controls. Specifically, several regions in the affected temporal lobe showed increased functional coupling with the ipsilateral insula and immediately neighboring subcortical regions. Additionally there was significantly decreased functional connectivity between regions in the affected temporal lobe and their contralateral homologous counterparts. Intriguingly, decreased local and inter-hemispheric connectivity was not limited or even maximal for the hippocampus or medial temporal region, which is the typical seizure onset region. Rather it also involved several regions in temporal neo-cortex, while also retaining specificity, with neighboring regions such as the amygdala remaining unaffected. These findings support a view of temporal lobe epilepsy as a disease of a complex functional network, with alterations that extend well beyond the seizure onset area, and the specificity of the observed connectivity changes suggests the possibility of a functional imaging biomarker for temporal lobe epilepsy.

## Highlights

• We studied functional connectivity changes in patients with temporal lobe epilepsy.
• Patients had *decreased local and inter-hemispheric* functional connectivity.
• Patients had *increased intra-hemispheric* connectivity, ipsilateral to seizure focus.
• Functional changes involved several neocortical sites, including the insula.
• This pattern may have usefulness as a non-invasive method for presurgical planning.

## 1 Introduction

Focal epileptic seizures arise from focal abnormal neuronal activity which can spread to other cortical regions (Jackson and Colman, 1898). Temporal lobe epilepsy (TLE) is a well-characterized example of focal epilepsy, with seizures that typically originate in the medial temporal region. From a structural/pathologic standpoint, TLE can often be associated with specific structural and metabolic abnormalities, including hippocampal atrophy and/or sclerosis (Hogan, 2001; Margerison and Corsellis, 1966; Van Paesschen et al., 1997) and medial temporal hypometabolism (Arnold et al., 1996; Carne et al., 2004; Theodore et al., 1992). Yet localized structural and/or metabolic abnormalities extend beyond the medial temporal region to multiple, non-limbic brain regions, most prominently lateral temporal and frontal regions, as noted via structural magnetic resonance imaging (Bernhardt et al., 2010), positron emission tomography (Arnold et al., 1996; Theodore et al., 1992), magnetic resonance spectroscopy (Miller et al., 2000; Stanley et al., 1998), and pathologic studies (Margerison and Corsellis, 1966). Furthermore several patients have normal brain MRI or PET scans, even after years of disease (Carne et al., 2004; O'Brien et al., 1997; Siegel et al., 2001; Smith et al., 2011), suggesting that gross structural abnormalities alone incompletely capture the disease process.

Focal epileptic seizures also often follow a stereotypical progression, with a sequence and appearance of clinical symptoms and signs which remains well conserved across seizures in an individual, and reflects the brain regions activated by the seizure (Wieser, 1983). This has a correlate at the metabolic level, noted with single photon emission tomography (SPECT) ictal perfusion imaging, with several brain regions becoming metabolically active during an epileptic seizure: in TLE, for instance, the medial temporal region is not the only region active at seizure onset, but lateral temporal regions, the insula, the thalamus, and the contralateral temporal lobe also are rapidly activated (Hogan et al., 2006).

These findings suggest that focal epileptic seizures reflect abnormalities that go beyond the seizure onset zone and involve a network of regions, and that epilepsy reflects network-level instability or pathology (Spencer, 2002). Indeed evidence exists, primarily from animal models, that seizures arise from cortico-cortical and cortico-subcortical networks, and can have stereotyped clinical appearance despite onset from different cortical foci (Bear et al., 1996; Spencer, 2002; White and Price, 1993). This supports the claim that the seizure onset zone belongs to a functional network, activation of which leads to a stereotyped clinical manifestation.

Functional connectivity measures derived from blood-oxygenation level dependent (BOLD) functional magnetic resonance imaging (MRI) characterize large-scale functional networks of brain regions in both healthy and patient populations. Functional network abnormalities have been associated with specific neurologic disorders (Buckner et al., 2009; Hawellek et al., 2011; He et al., 2007). While some studies in epilepsy noted decreases in functional connectivity localized near the seizure onset zone (Bettus et al., 2009, 2010; Pereira et al., 2010; Pittau et al., 2012; Zhang et al., 2010), potentially suggesting that the pathological changes brought on by the disease also result in functional network disruption, others showed regions of increased connectivity. Liao and colleagues used graph theory to examine functional connectivity in a broad set of brain regions in patients with TLE, noting significantly increased connectivity within the medial temporal lobes, but decreased connectivity within frontal and parietal regions (Liao et al., 2010). Bettus and colleagues noted increased interictal connectivity within the ictal zone by using surface EEG in a smaller group of TLE patients (Bettus et al., 2008). Morgan and colleagues noted disrupted connectivity between hippocampi of TLE patients, but with increasing connectivity with longer disease duration (Morgan et al., 2011). The range of findings reflect in part the nascent nature of the techniques used to analyze resting-state activity, including how one defines regions of interest to use as “seeds” in calculating functional connectivity with other brain regions, as well as the heterogeneity of the patient populations examined.

Here, we defined regions of interest in each patient with unilateral TLE based on their individual anatomy, focusing on structures involved, either structurally or metabolically, in TLE. Resting on a potential model of epilepsy as an abnormal functional network of brain regions, we hypothesized that regions that are typically involved in the onset and propagation of seizures in TLE, i.e. the medial and lateral temporal region ipsilateral to the seizure focus, would show abnormally increased connectivity. Based on the existing literature, we also hypothesized that connectivity changes in the ipsilateral temporal region would be associated with changes in resting state brain connectivity with other brain regions, including the contralateral temporal structures.

## 2 Methods

### 2.1 Participants

Thirty-two patients with temporal lobe epilepsy (20 with left TLE, 12 with right TLE) were retrospectively and consecutively enrolled through the Washington University Adult Epilepsy Center (Table 1). Patients were screened for enrollment based on reported clinical semiology of their seizures and confirmed *unilateral* temporal lobe epilepsy by video-EEG monitoring. Clinical semiology for study inclusion included auras of epigastric rising, experiential phenomena (most commonly fear) and gustatory or olfactory sensations (Hogan, 2001). Subjects with auras suggestive of lateral temporal onset seizures, including auditory hallucinations, visual misperceptions, or language disturbance, were excluded from the study group (Commission on Classification and Terminology of the International League Against Epilepsy, 1989). Typical ictal clinical signs of our patient group included arrested activity, oro-alimentary automatisms, decreased responsiveness, and motor automatisms which typically involved the contralateral upper extremity (Wieser, 1986). All subjects had complex partial seizures during the course of their video EEG monitoring. Ictal scalp EEG patterns showed focal anterior temporal distribution slowing either at EEG seizure onset, or showed progression to rhythmic focal slowing over the involved anterior temporal region in the majority of seizures (Risinger et al., 1989). Criteria for exclusion at any time during the study included clinical or electrographic evidence of bitemporal or extratemporal seizures, developmental anomalies, cortical malformations or other focal lesion on structural MRI, age < 18 years, contraindication to MRI, including suspected pregnancy, history of substance or alcohol abuse, and non-proficiency in the English language. A subset of the patients had MRI evidence of medial temporal sclerosis (as assessed via MRI image properties) and/or hippocampal atrophy, a common finding in TLE, but this was purposefully neither an inclusion nor an exclusion criterion. Importantly, none of the patients showed bilateral pathologic changes. A group of healthy control subjects that were individually matched in terms of age (+/− 2 years), gender and handedness to each TLE patient (n = 32) were studied under identical imaging conditions for comparison. The institutional review board at Washington University in St. Louis approved the study. All participants signed written consent to participate in the study.

### 2.2 MRI Acquisition and Preprocessing

Images were acquired with a Siemens MAGNETOM Trio 3 T scanner (Erlangen, Germany). A high-resolution (0.42 × 0.42 × 0.9 mm) T1-weighted MPRAGE (TI 800 ms, TE 3.29 ms) structural scan was obtained in each subject for the purpose of anatomic segmentation, registration to the functional images, and atlas transformation. Two BOLD resting state functional scans (echoplanar, TR 2200 ms, TE 27 ms, flip angle 90 deg, 4x4x4 mm voxels) were also acquired for each subject. Each functional run was comprised of 164 volumes (approximately 6 minutes in duration). Study participants were asked to relax while fixating on a cross hair. fMRI BOLD data was pre-processed in several steps using standard methods (Snyder, 1996). Motion was corrected with rigid-body realignment. Spurious variance was minimized by removal of the linear trend, temporal low-pass filtering, spatial smoothing, and regression of nuisance parameters (head-motion, white matter, ventricular and global signals) and their temporal derivatives (Fox et al., 2009).

### 2.3 Regions of Interest (ROIs)

Medial temporal ROIs were defined based on segmentation of each participant's high-resolution T1-weighted MRI scan. Automated volumetric segmentation was performed using the Freesurfer image analysis suite (version 4.5.0, https://surfer.nmr.mgh.harvard.edu) and yielded individualized volumes corresponding to given anatomic regions of interest, including hippocampus, parahippocampal gyrus, fusiform gyrus, inferior temporal gyrus, middle temporal gyrus, superior temporal gyrus, insula and amygdala bilaterally. This individualized method of ROI definition was chosen to minimize the contribution of non-tissue (e.g. CSF) to the regions of interest of participants with atrophy. Furthermore, it more reliably accounted for individual variability in the anatomic position and extent of these regions. The accuracy of the segmentation was verified visually for each subject, as well as quantitatively for hippocampal regions by quantifying hippocampal volume and by comparing the Freesurfer segmentation to a more accurate hippocampal shape assessment method via a high-dimensional large deformation algorithm (Hogan et al., 2003). Participant-specific anatomic regions defined in this fashion were used to extract BOLD functional data for use in subsequent analyses. Functional MRI BOLD time-series for the purpose of estimating functional connectivity strength of ROI pairs were extracted using ROIs in subject space (i.e. without transformation to atlas space). Images were resliced and transformed into a standard atlas space (Talairach and Tournoux, 1988) only when generating group connectivity maps.

### 2.4 Functional connectivity measures

Functional connectivity analyses used established methods based on Pearson product moment correlation between time-series (Fox et al., 2005). Correlation measures were computed both for *a priori* selected ROI pairs and in exploratory whole-brain analyses. For ROI–ROI analyses, the average BOLD time-series was extracted from each region and correlated region-by-region with the other ROIs, yielding a correlation matrix (Fox et al., 2005). For whole-brain functional connectivity analyses, time-series were extracted from ROIs and correlation maps were generated by computing the correlation with every voxel in the brain. Data were collapsed across patients at the group level into results representing the hemisphere ipsilateral and contralateral to the seizure focus.

### 2.5 Statistical analysis

All computed Pearson correlation coefficients (r) were first transformed using Fisher's z-transform prior to further analysis (Fox et al., 2009).

Group-level seed-based connectivity maps were generated for main effects and comparisons between controls and TLE patients, the latter combining L TLE and R TLE groups collapsed according to ipsilateral vs. contralateral to seizure onset. This was achieved by computing a voxel-wise t-test, and normalizing it to a Z-score and correcting for significant cluster size using a Monte Carlo-based estimation of noise thresholds derived from an independent set of healthy controls (similar to Bullmore et al., 1999; McAvoy et al., 2001).

For ROI pair analyses, in general, statistical models were designed to test the effect of disease on the dependent variable of functional connectivity strength between ROIs in a given pair (i.e. the Fisher's z-transformed correlation between the two ROI time-series). In a first ANOVA, factors of group (control vs. L TLE vs. R TLE) and ROI pair were included as independent variables. Secondly, for the purpose of maximizing potential differences between the two patient groups, the model was simplified to a second ANOVA identically designed to the first, but only containing data from the two patient groups (L TLE vs. R TLE).

In an additional analysis, done for the purpose of further testing potential effects of hemisphere and disease laterality, a new ANOVA was calculated with functional connectivity strength as the dependent variable, and disease (control vs. TLE), hemisphere (left or right), laterality (of the examined ROI pair) with respect to the seizure focus (ipsilateral vs. contralateral), and ROI pair as independent factors. The power of this analysis was improved by pairing each control to a given TLE patient based on the age, gender, and handedness matching. Only within-hemisphere ROI pairs were tested in this analysis to maximize potential effects of hemisphere (and not dilute a potential effect by including cross-hemisphere ROI pairs).

To generate a network connectivity map highlighting differences between TLE patients and controls, post-hoc paired t-tests were computed for individual ROI pairs. Pairing between controls and TLE patients was based on matching by age, gender and handedness as outline above.

## 3 Results

**Supplementary Fig. 1.**
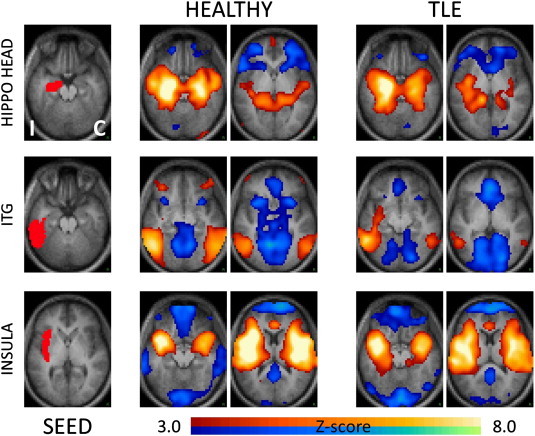
Seed-based correlation maps showing the group main effect for healthy controls and TLE patients. Representative seeds in the ipsilateral medial temporal (hippocampal head), lateral temporal (ITG) and insular regions are shown in the left column. The remaining columns show the Z-score maps at selected axial locations. As in prior figures, the hemisphere ipsilateral to the seizure focus is mapped on the left (I) while the contralateral hemisphere is mapped on the right (C). Illustrated clusters are significant (p < .01) after correction for multiple comparisons. Blue–green colors indicate negative correlations, red–yellow indicate positive correlations.

TLE appears to have a complex effect on the functional connectivity of mesial temporal and neocortical temporal regions. Whole-brain group connectivity maps in TLE patients showed overall strong functional connectivity for all seeds examined, which qualitatively reflected the connectivity patterns of their matched controls (Supplementary Fig. 1). However TLE patients showed significant quantitative differences from controls, as follows.

TLE appears to have a complex effect on the functional connectivity of mesial temporal and neocortical temporal regions. Whole-brain group connectivity maps in TLE patients showed overall strong functional connectivity for all seeds examined, which qualitatively reflected the connectivity patterns of their matched controls (Supplementary Fig. 1). However TLE patients showed significant quantitative differences from controls, as follows.

### 3.1 Medial temporal and neocortical temporal regions in TLE show decreased local coupling compared to controls

**Fig. 1:**
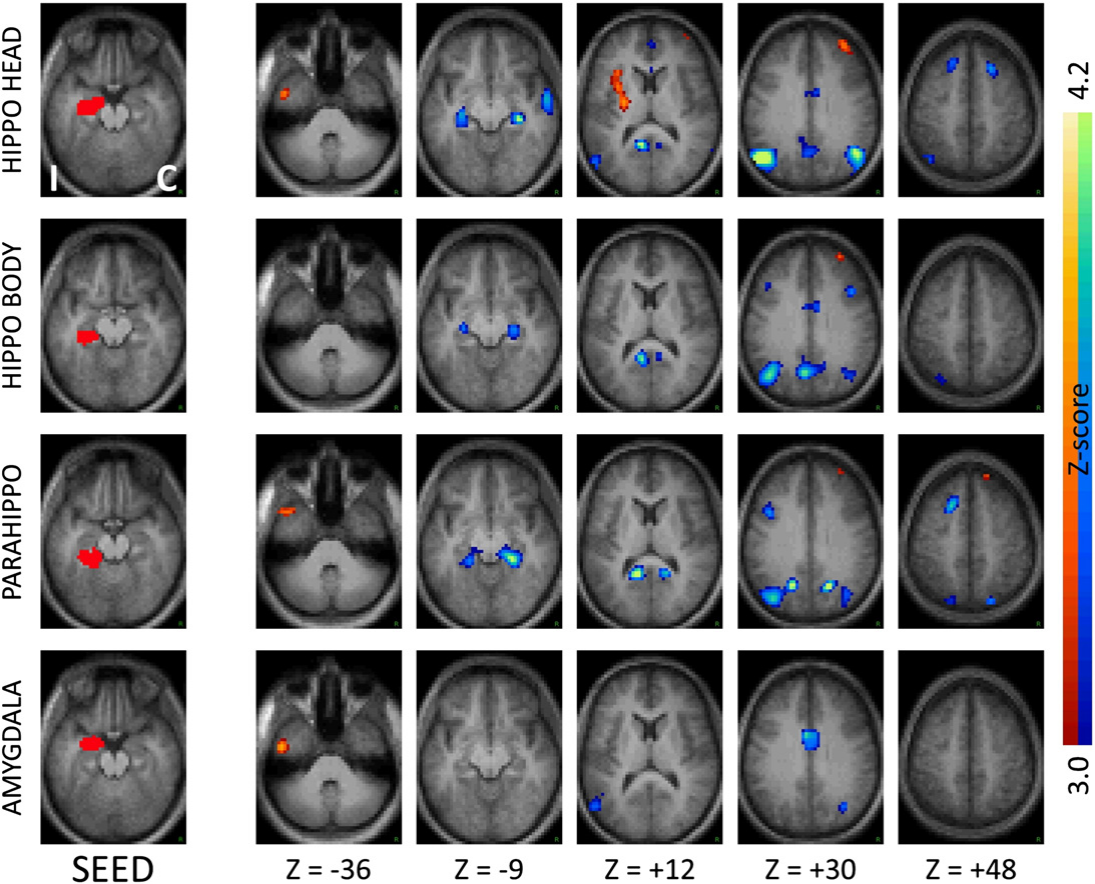
Seed-based correlation maps comparing TLE patients to controls for seeds in ipsilateral medial temporal regions. The left column shows the seed, while the remaining columns show the Z-score maps at selected axial slices. Slice labels are in Talairach coordinates (Talairach and Tournoux, 1988). The hemisphere ipsilateral to the seizure focus is mapped on the left (“I”) while the contralateral hemisphere is mapped on the right (“C”). Illustrated clusters are significant (p < .01) after correction for multiple comparisons. Blue–green colors indicate decreased correlations in patients, red–yellow indicate increased correlations. Ipsilateral hippocampal head, body and parahippocampus but not amygdala show significant functional decoupling with their contralateral homologues in TLE patients. The ipsilateral hippocampal head also shows *increased* functional coupling with the ipsilateral insula.

**Fig. 2:**
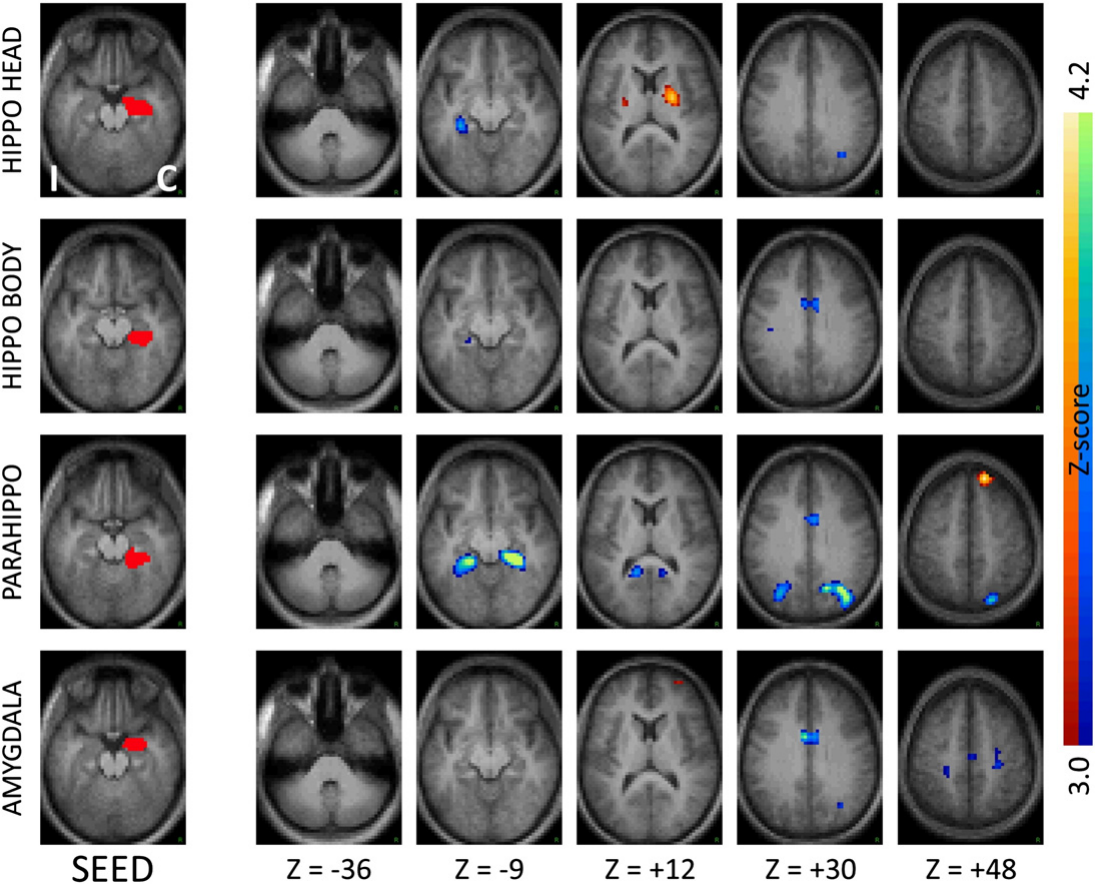
Seed-based correlation maps as in Fig. 1 comparing TLE patients to controls for seeds in contralateral medial temporal regions. Note the decreased functional connectivity with the ipsilateral medial temporal regions, mirroring the effects seen with the ipsilateral seeds.

**Fig. 3:**
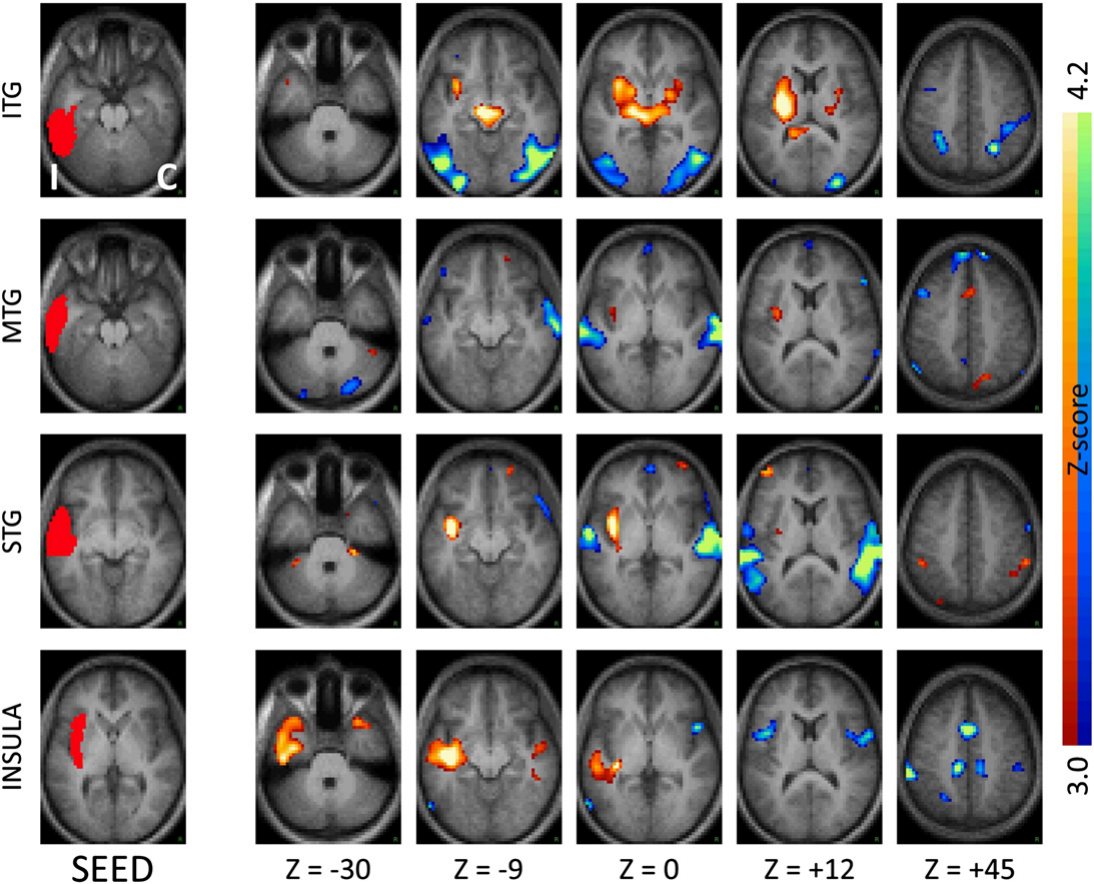
Seed-based correlation maps as in Fig. 1 for seeds in ipsilateral neocortical temporal regions and insula. Note the significantly reduced inter-hemispheric correlations, and significantly increased coupling with the ipsilateral insula and immediately neighboring subcortical regions. ITG — inferior temporal gyrus, MTG — middle temporal gyrus, STG — superior temporal gyrus.

**Fig. 4:**
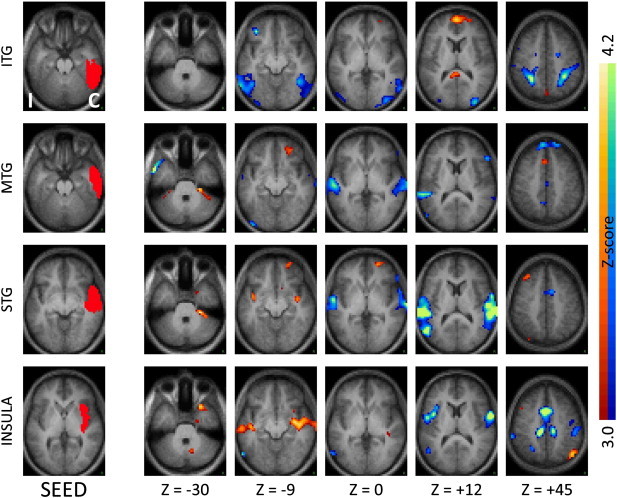
Seed-based correlation maps as in Fig. 1 comparing TLE patients to controls for seeds in the contralateral neocortical temporal regions and insula. Note the decreased functional connections with the ipsilateral lateral temporal regions, mirroring the effects seen with the ipsilateral seeds.

The coupling of seeds in both medial temporal and neocortical temporal regions with local neighboring voxels was significantly decreased (paired t-test, p < .01) compared to controls (see Figs. 1–4, rows 1–3). This was evident for seeds in the hippocampus, parahippocampus, and neocortical temporal lobe (superior, middle and inferior temporal gyrus). It appeared most pronounced for seeds ipsilateral to the seizure focus, but was also present for seeds in the contralateral hemisphere. This phenomenon of local decoupling is reminiscent of and may be related to the regions of atrophy, cortical thinning or local hypometabolism that can often be seen in TLE patients (Arnold et al., 1996; Bernhardt et al., 2009).

### 3.2 Specific medial temporal and neocortical temporal regions in TLE are functionally decoupled from their contralateral counterparts

In addition to effects of local functional decoupling, inter-hemispheric functional connectivity was also significantly reduced in TLE (paired t-test, p < .01) compared to controls (Figs. 1–4, first three rows), suggesting that both medial temporal and neocortical temporal regions become relatively decoupled in the disease. In the medial temporal region, areas of decreased functional connectivity closely followed the anatomic distribution of the bilateral hippocampal formations, and continued posteriorly into the bilateral parahippocampi and bilateral posterior cingulate regions. Notably, and somewhat expectedly given that the seeds chosen overlapped with homologous regions in the two hemispheres, this effect was most pronounced for regions ipsilateral to the seizure focus (Fig. 1, first three rows) but also present for regions contralateral to the seizure focus (Fig. 2, first three rows). The effect of TLE on temporal functional networks extended beyond the medial temporal areas typically implicated in seizure onset. Specifically, seeds in the inferior temporal, middle temporal and superior temporal gyrus all showed significant levels of functional decoupling with their contralateral counterparts (Figs. 3 and 4, first three rows), relatively similar in seeds both ipsilateral and contralateral to the seizure focus.

Importantly, the effects of local and interhemispheric decoupling noted above were not universal for the entire temporal region, but were specific to particular locations. An example of this specificity was observed when the amygdala was used as a seed. This area is immediately adjacent to the hippocampal head anatomically, and yet did not show the local and interhemispheric functional decoupling noted for the other medial temporal seeds (Fig. 1, bottom row).

Although not formally explored in this study, the effect of TLE was not limited to the temporal region. For instance, the medial temporal region ipsilateral and contralateral to the seizure focus also showed decreased functional connectivity with specific extratemporal brain regions: the hippocampal head, body and parahippocampus all showed significantly reduced correlations with regions in bilateral lateral parietal, medial parietal and medial frontal cortex (Figs. 1–2, first three rows).

### 3.3 TLE patients exhibit an abnormal network of facilitated functional connectivity in the affected temporal lobe, spanning the temporal archicortex, temporal neocortex and insular regions

Several of the temporal regions that showed functional decoupling with their respective homologous counterparts in the contralateral hemisphere also showed concurrently *increased* functional coupling with the ipsilateral insula and neighboring subcortical regions, demonstrating the complexity of reorganization of functional networks in focal epilepsy. As shown in Fig. 1 (first row) the ipsilateral hippocampal head, a typical seizure onset region in TLE, showed increased functional connectivity with the ipsilateral insula. Neocortical temporal regions such as the inferior temporal gyrus, superior temporal gyrus, and to a lesser extent the middle temporal gyrus, also showed increased functional coupling with the ipsilateral insula as well as immediately neighboring subcortical regions (Fig. 3, first three rows). In confirmatory fashion, a seed placed in the ipsilateral insula showed reciprocal effects (Fig. 3, bottom row). The effects of increased facilitation in connectivity between temporal regions and ipsilateral insula were most prominent for the hemisphere ipsilateral to the seizure focus, though weak effects were also seen in the contralateral hemisphere (Figs. 2 and 4).

To further explore these observations made on connectivity maps, a statistical analysis was performed testing the functional connectivity of ROI pairs, using as ROIs the seeds utilized in the previous analyses and the Fisher z-transformed Pearson correlation between ROIs as the dependent variable. A first ANOVA, with factors of group (control vs. L TLE vs. R TLE) and ROI pair, yielded a significant effect of ROI pair (F = 79.2, p < .001) and a significant interaction of ROI pair with group (F = 2.1, p < .001), with no significant effect of group (F = 0.75, p = 0.49).

The approach of collapsing patients with left TLE and right TLE into the same group, done for the purpose of investigating effects with regards to co-laterality with the seizure focus, may lead to confounds if the patient groups differ or if the hemispheres differ with respect to the connectivity of the brain regions examined. We thus conducted additional confirmatory analyses to specifically address these potential confounds.

To further investigate a potential difference between left TLE and right TLE groups, the same ANOVA as above was repeated without including the control group (i.e. only left TLE and right TLE groups included, with the goal of maximizing a potential effect of group due to TLE group differences). This yielded a significant effect of ROI pair (F = 31.3, p < .001), but no significant effect of group (F = 0.16, p = 0.69) and no significant interaction of group with ROI pair (F = 0.71, p = p = 0.99), suggesting that there was no significant difference between the patient groups.

To further explore whether an effect of hemisphere or disease laterality confounded the results a third ANOVA was performed. Factors included group (control vs. TLE), hemisphere (left or right), disease laterality (of the examined ROI pair) with respect to the seizure focus (ipsilateral vs. contralateral), and ROI pair. To ensure even weighting of the analysis, a control was matched to each patient. Only within-hemisphere ROI pairs were tested in this analysis to avoid diluting a potential effect of hemisphere by including cross-hemisphere ROI pairs. Significant main effects included group (control vs. TLE; F = 7.6, p < .001) and ROI pair (F = 63.8, p < .001). Significant interactions included group by disease laterality (F = 2.3, p < .05) and group by ROI pair (F = 1.8, p < .001). There were no other significant effects or interactions. Notably, there were no significant effects of hemisphere, neither through a main effect (F = 0.68, p = 0.41) or via interactions (hemisphere by group, F = 2.34, p = 0.13, hemisphere by pair, F = 0.77, p = 0.92, or hemisphere by group by pair, F = 1.04, p = 0.38), suggesting that for the purpose of the ROI pairs examined, hemisphere was not a significant factor.

Also of note, while there was no main effect of disease laterality (i.e. whether a given ROI pair was ipsilateral or contralateral to the seizure focus), there was a weak interaction of group with disease laterality. To explore this interaction a fourth ANOVA structured similarly to the third was calculated, leaving out controls. This showed a weakly significant main effect of laterality (F 4.21, p < .05) and a significant main effect of ROI pair (F = 28.55, p < .001), without any other significant main effects (hemisphere, F = 0.24, p = 0.62) or interactions (hemisphere by pair, F = 0.70, p = 0.97). These findings suggest that for TLE patient laterality of a given ROI pair with respect to seizure focus played a small but significant role, possibly reflecting the increased connectivity noted for specific seeds ipsilateral to the seizure focus in the connectivity maps described above.

**Fig. 5:**
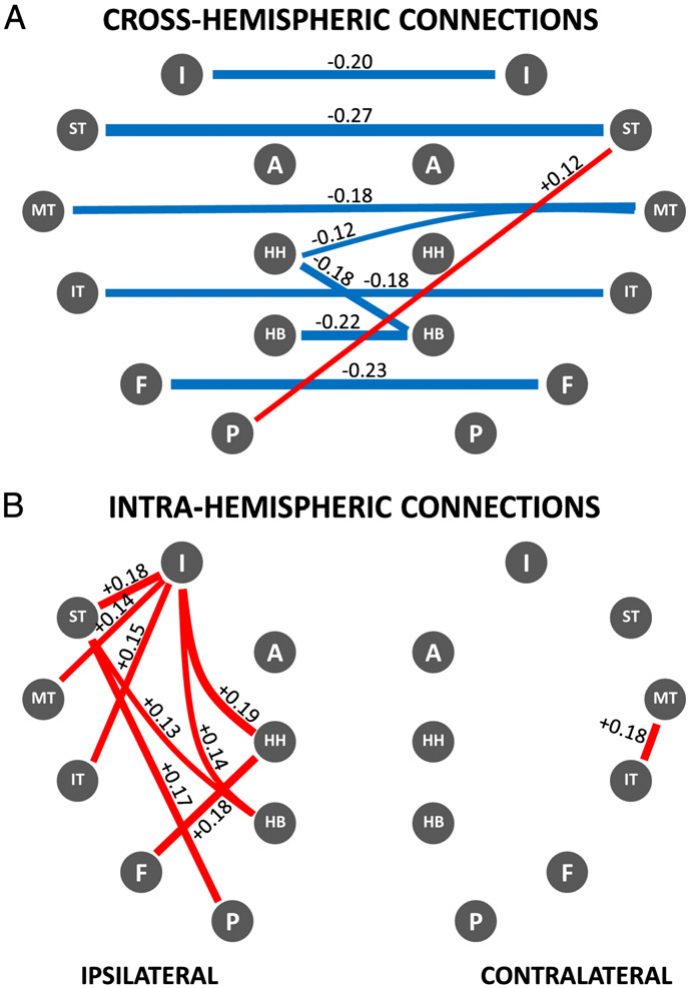
Network map of correlation differences between TLE patients and controls — A: cross-hemispheric connections; B: intra-hemispheric connections. Blue lines represent significantly decreased correlations in patients compared to controls; red lines represent significantly increased correlations in patients compared to controls (p < .01). Strength of the difference in coupling between patients and controls is indicated numerically next to each connecting line as difference between correlation strengths as well as by the relative line thickness. Note significant functional decoupling across hemispheres that affects both medial and lateral/neocortical temporal regions. Note increased correlations in the temporal region ipsilateral to the seizure focus, especially involving the insula. Legend: HH: hippocampal head, HB: hippocampal body, P: parahippocampus, F: fusiform gyrus, IT: inferior temporal gyrus, MT: middle temporal gyrus, ST: superior temporal gyrus, I: insula, A: amygdala.

To further test these results, post-hoc t-tests of individual ROI pairs were conducted that reinforced the observations made above. For ease of visualization the results are reported in the form of a network map showing differences in connectivity strengths in patients compared to controls (Fig. 5). Results demonstrated significant decoupling of both medial and neocortical temporal regions with their contralateral counterparts and increased functional connectivity of those regions with the ipsilateral insula, reflecting the findings derived with the connectivity maps. Increased coupling was largely confined to the ipsilateral temporal region, with minimally increased functional connectivity in the contralateral hemisphere.

## 4 Discussion

We investigated the resting state BOLD functional connectivity of the temporal region in TLE, in patients with well-established clinical semiology and seizure onset. Our findings demonstrate that the disease has profound effects on the functional network of the temporal lobe that extend beyond the hippocampus and medial temporal region. Importantly, the observed functional network disruption targets a specific set of regions which mirrors the propagation pattern of temporal seizures (Hogan et al., 2006), providing a functional correlate of the disease.

### 4.1 TLE leads to local functional decoupling of regions in the bilateral medial temporal lobe and temporal neocortex

Several regions of the temporal lobe including the hippocampus, parahippocampus, and superior, middle and inferior temporal gyrus showed significantly reduced connectivity with voxels immediately neighboring and overlapping the seed of interest, as compared to healthy controls. These results replicate and extend findings of decreased connectivity near the epileptogenic zone and in the neighboring medial temporal region observed in other studies of functional connectivity in TLE (Bettus et al., 2009, 2010; Mankinen et al., 2012; Pereira et al., 2010; Pittau et al., 2012). Such local effects are reminiscent of and seem to co-localize with regions of hypometabolism that are often seen with positron emission tomography in patients with TLE (Arnold et al., 1996). It seems probable that local neuronal loss, especially in the case of the medial temporal region, may play at least a partial role both in the historically observed patterns of hypometabolism and in our findings of locally decreased functional connectivity, though the extent of this contribution is presently unknown. Furthermore, even in a scenario without cell loss where neurons are preserved but show pathologic function, functional connectivity of “sick” neurons is likely to be impaired and diminished compared to healthy neurons. Also notable was the finding that even after carefully selecting a population of patients with unilateral temporal seizures, the effect of local decoupling was present, though in less prominent fashion, even for temporal regions contralateral to the seizure onset zone. This can be interpreted as a functional confirmation of the often observed finding of bilateral temporal hypometabolism in patients with unilateral temporal seizures (Carne et al., 2004; Henry et al., 1993; O'Brien et al., 1997), though significantly asymmetric medial temporal hypometabolism is associated with greater rates of postoperative seizure freedom (Boling et al., 2008).

### 4.2 TLE leads to cross-hemispheric functional decoupling of the temporal lobes

A main observation of this study was reduced inter-hemispheric functional connectivity in TLE patients affecting medial and neocortical/lateral temporal regions. A few previous studies of functional connectivity in TLE have focused on intrahemispheric effects (Bettus et al., 2009, 2010), while others have noted decreased connectivity between medial temporal regions (Pittau et al., 2012), but typically without extending the scope to the temporal neocortex. As with local changes described above, decoupling of the medial temporal region at the functional level may in part reflect the cellular pathophysiologic changes observed in some TLE patients manifesting as hippocampal atrophy and mesial temporal sclerosis (Hogan, 2001), some of which reflects neuronal cell loss. In our study, we defined regions of interest based on the individually segmented anatomy of each participant, with the specific objective of reducing the effect of atrophy on measured functional coupling. Thus, voxels that did not contain brain parenchyma were not included in the computation. However this method does not account for small-scale pathological changes not captured by the image resolution of the segmentation technique, which is in the millimeter range (Smith et al., 2011). Intriguingly, the inter-hemispheric functional decoupling noted in homologous medial temporal regions was mirrored by neocortical sites, including the inferior, medial and superior temporal lobes, all of which showed reduced functional connectivity with their homologues in the contralateral hemisphere. This finding may still reflect a degree of neuronal loss similar to that observed in the medial temporal region, a speculation already supported by some studies (Voets et al., 2012). Yet minimal, if any, thinning in lateral temporal neocortical/non-limbic regions has been reported in TLE patients (Bernasconi et al., 2004; Bernhardt et al., 2009). Alternatively, reduced inter-hemispheric BOLD correlations may reflect true network-level changes in intrinsic neural activity. If such changes are indeed present in TLE, it is unclear whether this represents adaptation, i.e. an attempt to protect the healthy, contralateral temporal lobe from the negative effect of temporal lobe seizures, or a maladaptive network-level manifestation of the disease process. For instance, loss of interhemispheric connectivity in stroke is associated with deficits in attention (He et al., 2007) and motor function (Carter et al., 2010).

### 4.3 TLE is associated with increased coupling within the temporal lobe ipsilateral to the seizure focus

A fundamental finding of this study is that several temporal regions (medial cortical and neocortical/lateral cortical) that showed functional decoupling with their contralateral homologues, concurrently showed increased functional connectivity ipsilateral to the seizure focus. This effect was well localized to the hemisphere of seizure origin, as compared to the contralateral temporal lobe, which showed similar, but much weaker effects.

It is noteworthy that the regions showing increased coupling (temporal and insular cortex and neighboring subcortical regions) have been previously identified as typically recruited by temporal lobe seizures (Hogan et al., 2006; Wieser, 1983). The presently observed increased functional coupling may thus represent an *interictal* functional correlate of an *ictal* seizure propagation pathway. If so, this would be a novel finding, in some ways complementary to the pathophysiologic correlate of mesial temporal sclerosis with location of seizure onset.

Particularly compelling is the fact that certain regions showed both increases and decreases in functional coupling, depending on the connection examined: such a result is not necessarily reducible to an effect of neuronal cell loss in the structures studied. Indeed both gross and microscopic cell loss is observed in the medial temporal region (and to a less degree in lateral temporal and extratemporal regions) in TLE. Due in part to the novel nature of the technique, the effect of cell loss on the functional connectivity of a brain region remains unclear. In one scenario cell loss could result in reduced signal-to-noise of a given region, because of the reduced number of functioning neurons, leading to correspondingly decreased correlation with the signal from other brain regions. In an alternative scenario, reduction via cell loss in the number of neurons contributing to the signal from a given region may paradoxically result in increased internal coherence of the remaining group: the activity of this more homogenous group of neurons, possibly reflective of a specific cell population, may then show improved correlation with selected other brain regions (Bonilha et al., 2012). Further studies that directly address this issue are needed.

As previously mentioned, regions involved in seizure onset have often been shown to have abnormally low metabolism (Arnold et al., 1996; Carne et al., 2004; Theodore et al., 1992). In the speculative scenario introduced above, the overall signal from the region is reduced (because of cell loss, possibly from a specific cell population), which would correlate with local hypometabolism, but the intrinsic coherence of the remaining functioning neurons is increased, leading to increased coupling with specific other brain regions. Reflecting the novel nature of this imaging technique, the relationship between functional connectivity and local metabolism is currently not well understood and should be addressed in future studies.

### 4.4 The insula (and its neighboring subcortical regions) as a TLE network hub

The insula ipsilateral to the seizure focus was a prominent locus of increased functional connectivity in TLE patients (Fig. 3), involving both medial connections with the ipsilateral hippocampus and neocortical connections with the ipsilateral inferior, middle and superior temporal gyri. Our findings suggest a role for the insula in the pathophysiology of TLE. The insula is generally thought to play a complex, integrative role in cognition, informed in part by the type of connections it receives: its posterior portion receives reciprocal somatosensory, nociceptive, thermal and visceral inputs, while its anterior aspect receives afferents from prefrontal and limbic regions (Mesulam and Mufson, 1982). Reflecting its connections, the insula has been implicated in modulating awareness and perception of the self through increasingly complex levels of integration in a posterior-to-anterior organization. The posterior insula appears to be involved at more basic levels of interoceptive perception, such as awareness/monitoring of one's body temperature, heartbeat, and other somatosensory/visceral information, while more anterior portions are devoted to an increasingly higher level of computations, including self-recognition, emotional awareness, risk assessment, decision making, and time perception (Craig, 2009; Critchley et al., 2004). The type of perceptual experiences reported by patients during temporal lobe seizures, which include visceral sensations, palpitations, time dilation, out-of-body experiences, etc. have remarkable overlap with the type of information modulated by the insula, leading investigators to implicate this region as a symptomatic zone during propagation of TLE seizures (Wieser, 1983). Our finding of increased insular coupling with multiple temporal regions is suggestive of a facilitated pathway in TLE involving this structure, possibly but not necessarily reflecting a pathway of seizure propagation. Indeed the insula may represent a facilitated network hub in TLE, whose role is better captured by a technique such as functional MRI, which, given the insula's deep location and overlay of prominent blood vessels, has an advantage over both scalp and cortical surface EEG. Despite technical obstacles, Isnard and colleagues performed depth electrode recordings of the insula demonstrating insular cortex involvement in all of 81 recorded TLE seizures, confirming the extremely common ictal involvement of the insula (Isnard et al., 2000). If the increased connectivity represents a facilitated pathway of propagation, this may manifest even interictally, potentially consistent with the increased connectivity of this region during interictal epileptiform discharges noted in a recent combined EEG–fMRI study (Fahoum et al., 2012). Also notable in our current study, in addition to increased functional connectivity to the insula, the inferior temporal gyrus also showed increased coupling with subcortical thalamic and basal ganglia regions, extending the potential network of facilitation in TLE. Indeed, thalamic and basal ganglia regions activate during intracranial EEG recordings of temporal lobe seizures (Rektor et al., 2002) and increased connectivity with the basal ganglia has been observed in generalized epilepsy (Luo et al., 2012). Additionally, ictal SPECT studies show activation in the ipsilateral anterior medial temporal, striatal, and insular regions during TLE seizures (Hogan et al., 2011), which correlates well with the ipsilateral regions of increased connectivity in the current study.

### 4.5 Increased temporal functional coupling as a candidate biomarker of seizure lateralization

A compelling finding of this study was the degree of lateralization ipsilateral to the seizure focus exhibited by the increased functional connectivity in the temporal network described above (Fig. 5). While not explicitly tested in this study, increased functional connectivity of the temporal region ipsilateral to the seizure focus has the potential to serve as a biomarker of TLE, with important and straightforward applications in treatment planning. As noted previously, a significant percentage of TLE patients do not have gross structural abnormalities on MRI or functional abnormalities on PET, making epilepsy surgery less successful (Siegel et al., 2001; Smith et al., 2011). Functional MRI detection of increased intra-hemisphere functional connectivity ipsilateral to the seizure focus may provide a non-invasive, well tolerated method of seizure lateralization or localization which can enhance presurgical planning, with the potential to improve surgical outcome (Stufflebeam et al., 2011), though the detection in our study of weaker effects in the contralateral hemisphere (Figs. 2 and 4) may temper the applicability of these findings.

### 4.6 Study limitations

In this study we used resting-state functional connectivity MRI to assess functional network changes in patients with TLE. The significance of resting-state functional connectivity changes is still being characterized even at the level of normal brain function, with some speculating that they represent a functional MRI correlate of the slow cortical potential (He et al., 2008). Some of the resting state networks described in normal subjects mirror groups of regions active during cognitive tasks, suggesting that the resting state activity has functional validity that reflects the network of connections used for specific cognitive functions (Biswal et al., 1995; Gusnard and Raichle, 2001; Lewis et al., 2009; Lowe et al., 2000; Peltier and Noll, 2002). Other resting state networks have also been described that do not clearly map to groups of regions engaged by specific cognitive functions. In these networks activity is in fact more pronounced when the brain is *not* engaged in a cognitive task. This led some to suggest that these groups of regions are not actual networks but instead reflect baseline brain physiology independent of anatomical or functional connections (Birn et al., 2006; Obrig et al., 2000; Wise et al., 2004). Others have speculated that they do reflect specific neuronal networks engaged when goal-directed action and external input are absent, i.e. “default” networks (Damoiseaux et al., 2006; De Luca et al., 2006; Gusnard and Raichle, 2001; Peltier and Noll, 2002; Raichle et al., 2001). Therefore, while the technique of resting state functional connectivity has been used in recent years to study a number of neurologic diseases (Buckner et al., 2009; Hawellek et al., 2011; He et al., 2007), it represents a recent approach to studying brain function, and conclusions from its application in patient populations should generally be derived with caution.

The majority of the analyses performed in this study collapsed left TLE and right TLE patients into a single patient group for the purpose of classifying effects as ipsilateral or contralateral to the seizure onset region. At first glance, given the numerous examples of asymmetry in the structural and functional connections of the human brain (Büchel et al., 2004; Saenger et al., 2012; Tomasi and Volkow, 2011) or the somewhat different patterns of seizure propagation of patients with left or right TLE (Hogan et al., 2006), this may have represented a confounding factor. However our confirmatory analyses accounting for patient group and hemisphere failed to reveal any additional significant effects, leading us to speculate that at least at the level of the large effects noted in this study the approach of combining patient groups was generally sound. Furthermore other studies of functional connectivity in TLE have noted substantial similarities in the connectivity pattern of the medial temporal region across these two patient groups (e.g. Morgan et al., 2011). However differences between left TLE and right TLE have also been noted. For instance Morgan and colleagues proposed using the connectivity of the right hippocampus with the ventral lateral nucleus of the right thalamus as a discriminant between left TLE and right TLE patients (Morgan et al., 2012). James and colleagues noted that while in right TLE patients the posterior cingulate gyrus shows functional disconnection with the right hippocampus, in left TLE patients the connectivity of this structure is reduced with both hippocampi (James et al., in press). Meaningful differences thus likely do exist in the connectivity of patients with left TLE vs. right TLE, but often have not been completely elucidated due to low power. Indeed, in our study of 32 patients it should be noted that while the effects and interactions of hemisphere or patient group were not significant, a weak but non-significant interaction of hemisphere by group (control vs. TLE) was seen in one analysis, suggesting that the connectivity asymmetry noted in previous studies may reflect an interplay between an intrinsic asymmetry in the connectivity of the human temporal lobes and temporal lobe epilepsy as a disease. This potentially crucial interaction should be better explored in future studies of greater statistical power.

Our study included subjects with ictal semiology characteristic of mesial temporal lobe epilepsy, confirmed with video EEG monitoring and showing ictal scalp EEG changes which localized to the involved temporal lobe. While scalp EEG recordings correctly predict findings of other techniques, such as intracranial electrode studies, approximately 90% of the time (Risinger et al., 1989), ictal scalp EEG patterns can be misleading in a minority of cases. For instance, as noted above, in a series of 21 patients who underwent stereotactic recordings from the insula, while 19 of 21 patients showed involvement of the insula at some point during the seizure, two of these patients (~ 10%) showed *ictal onset* in the insula (Isnard et al., 2000). It is thus possible that nodes in the network of increased connectivity described in this study which are outside the hippocampus, including the insula, may have functioned as the *ictal onset zone* and not just as a region of secondary propagation of the seizure. Similarly, ictal EEG semiology for complex partial seizures, while predictive of the region of origin of seizures, is also not completely specific for seizure localization (Palmini and Gloor, 1992). In addition, it has been historically difficult to distinguish between medial and lateral temporal onset seizures using only clinical semiology and ictal scalp EEG. While there are a number of clinical and electrographic differences between subjects with mesial and lateral onset TLE, they are not sufficient to allow definitive distinction in all subjects (O'Brien et al., 1996). Indeed, this may also reflect the heterogeneity noted in our group with regards to the presence of hippocampal atrophy. Yet recent studies have noted widespread connectivity differences involving the medial temporal region even in patients with non-lesional epilepsy (Weaver et al., 2013). However despite the possibly heterogeneous origin of seizures (i.e. mesial vs. lateral temporal onset) in some of our subjects, the overall findings of increased ipsilateral hemispheric connectivity and decreased local and inter-hemispheric connectivity remain as robust results in our otherwise homogeneous study cohort. Furthermore our group of patients is reflective of the population of patients with temporal lobe seizures that are routinely seen in adult epilepsy care, supporting the generalizability of our findings. Further studies to evaluate specific subgroups of subjects with TLE will likely be helpful to understand the interaction between resting state functional network changes and medial and lateral TLE.

### 4.7 Conclusion

We performed resting-state fMRI in thirty-two well localized TLE patients. Our principal findings are decreased local and inter-hemispheric functional connectivity and increased intra-hemispheric functional connectivity ipsilateral to the seizure focus. These findings have implications for the understanding of interictal pathophysiology of the temporal lobe and surrounding structures in patients with TLE, provide evidence that TLE is a disease of network dysfunction, and may have applications in both the diagnosis and treatment of TLE.

The following are the supplementary data related to this article.

Supplementary data to this article can be found online at http://dx.doi.org/10.1016/j.nicl.2013.06.011.

## Figures and Tables

**Table 1:** Patient demographic and clinical data.

Subject	Age/gender	Handedness	Age at onset	EEG	HA	Disease duration
*Left TLE*						
LTLE01	26/M	R	5	L TL	Y	24
LTLE02	45/F	R	32	L TL	N	13
LTLE03	59/M	R	48	L TL	N	12
LTLE04	37/F	L	22	L TL	Y	22
LTLE05	19/F	R	1	L TL	N	18
LTLE06	35/F	R	14	L TL	Y	21
LTLE07	64/F	R	25	L TL	N	39
LTLE08	51/F	R	23	L TL	Y	28
LTLE09	34/F	R	14	L TL	Y	20
LTLE10	32/F	R	8	L TL	N	24
LTLE11	54/M	L	48	L TL	N	6
LTLE12	43/F	R	38	L TL	N	2
LTLE13	30/M	R	24	L TL	N	15
LTLE14	32/M	L	3	L TL	Y	29
LTLE15	53/M	R	38	L TL	N	15
LTLE16	57/M	R	42	L TL	Y	16
LTLE17	45/F	R	42	L TL	N	3
LTLE18	20/M	R	19	L TL	N	1
LTLE19	65/F	R	63	L TL	N	6
LTLE20	46/M	L	43	L TL	Y	3

*Right TLE*						
RTLE01	55/F	R	3	R TL	Y	52
RTLE02	43/F	R	36	R TL	Y	7
RTLE03	40/M	R	1	R TL	Y	40
RTLE04	24/F	R	1	R TL	Y	24
RTLE05	42/F	R	41	R TL	N	2
RTLE06	27/F	R	10	R TL	Y	17
RTLE07	57/M	R	8	R TL	Y	46
RTLE08	32/F	R	1.5	R TL	N	31
RTLE09	49/F	R	14	R TL	Y	26
RTLE10	31/F	R	5	R TL	Y	26
RTLE11	69/M	R	4	R TL	N	65
RTLE12	40/F	R	31	R TL	N	9

EEG: seizure locus based on EEG; Disease Duration: time interval, in years, between first seizure and MRI scan; Legend: M — male, F — female, L — left-handed, R — right-handed, TL — temporal, HA — hippocampal atrophy. Note: patients with pathologic changes only showed changes ipsilateral to the seizure zone, based on MRI.
